# Effect of Digestate and Straw Combined Application on Maintaining Rice Production and Paddy Environment

**DOI:** 10.3390/ijerph18115714

**Published:** 2021-05-26

**Authors:** Xue Hu, Hongyi Liu, Chengyu Xu, Xiaomin Huang, Min Jiang, Hengyang Zhuang, Lifen Huang

**Affiliations:** 1 Jiangsu Key Laboratory of Crop Genetics and Physiology/Jiangsu Key Laboratory of Crop Cultivation and Physiology, Agricultural College of Yangzhou University, Yangzhou 225009, China; hx1997x2021@163.com (X.H.); nliuhy@nercita.org.cn (H.L.); Simonxcy@163.com (C.X.); hxm2020@yzu.edu.cn (X.H.); Jiangmin@yzu.edu.cn (M.J.); hyzhuang@yzu.edu.cn (H.Z.); 2 Jiangsu Co-Innovation Center for Modern Production Technology of Grain Crops, Yangzhou University, Yangzhou 225009, China

**Keywords:** digestate, straw, paddy soil, environmental effect

## Abstract

Few studies have focused on the combined application of digestate and straw and its feasibility in rice production. Therefore, we conducted a two-year field experiment, including six treatments: without nutrients and straw (Control), digestate (D), digestate + fertilizer (DF), digestate + straw (DS), digestate + fertilizer + straw (DFS) and conventional fertilizer + straw (CS), to clarify the responses of rice growth and paddy soil nutrients to different straw and fertilizer combinations. Our results showed that digestate and straw combined application (i.e., treatment DFS) increased rice yield by 2.71 t ha^−1^ compared with the Control, and digestate combined with straw addition could distribute more nitrogen (N) to rice grains. Our results also showed that the straw decomposition rate at 0 cm depth under DS was 5% to 102% higher than that under CS. Activities of catalase, urease, sucrase and phosphatase at maturity under DS were all higher than that under both Control and CS. In addition, soil organic matter (SOM) and total nitrogen (TN) under DS and DFS were 20~26% and 11~12% higher than that under B and DF respectively, suggesting straw addition could benefit paddy soil quality. Moreover, coupling straw and digestate would contribute to decrease the N content in soil surface water. Overall, our results demonstrated that digestate and straw combined application could maintain rice production and have potential positive paddy environmental effects.

## 1. Introduction

China is the largest consumer and producer and a major importer of chemical fertilizer [1]. In spite of its vital importance for food security, the overuse of chemical fertilizer has caused environmental problems globally, such as greenhouse gas (GHG) emissions and groundwater pollution [2]. As renewable resources with high efficiency and rich nutrients, straw and digestate have a great potential to substitute chemical fertilizer and promote sustainable agriculture development in China [3,4].

Agricultural wastewater, such as husbandry wastewater, aquaculture wastewater and liquid digestate, is easy to collect and utilize. The valuable resources in agricultural wastewater should be recycled and reused for environmental sustainability. Digestate is a kind of residue liquid of biogas produced by anaerobic fermentation with livestock and poultry manure as the main raw materials [5]. Valorization of livestock and poultry manure by anaerobic digestion has been considered as a standout option for bioenergy production in terms of energy efficiency and environmental impact [6]. Digestate can act as soil conditioner and provide valuable nutrients to plants [7,8], or digestate can be directly used as a nutrient source for soilless cultivation [9]. However, it is more often widely used as fertilizer in crop production [10,11]. With abundance in nitrogen (N), phosphorus (P), organic matter, trace elements and a variety of hydrolases, digestate is considered as a high-quality fertilizer to increase crop yield and improve soil quality [12,13]. Tang found that after applying digestate, rice grain yield was 7.48 t ha^−1^, which was 8.9% higher than that resulting from conventional fertilization [14]. As a liquid fertilizer, digestate can not only meet the water and nutrient demands for rice growth, but also reduce the risk of environmental pollution caused by agricultural wastes [15].

As another agricultural waste, straws are rich in cellulose, hemicellulose, lignin and other carbon compounds [16]; straw production in China accounts for ~25% of the world’s total straw resource [17] and straw incorporation is considered a potential approach to improve soil fertility and even boost rice production [18,19,20]. Previous studies found that straw return could improve soil organic matter (SOM) and structural stability during degradation processes, leading to a better soil function [21,22,23]. Moreover, several studies showed that straw addition could increase the activities of soil enzymes, including urease, phosphatase and catalase [24,25]. However, previous studies mainly focused on digestate and straw addition separately. The impacts of the combined application of digestate and straw on crop production and soil properties are still unclear. Therefore, it is of vital importance to know the effect of the combined application of digestate and crop residue on rice production and the paddy environment.

By 2050, the global demand for rice (*Oryza sativa* L.) is expected to increase by 28% [26]. China is the largest rice producer in the world, accounting for more than 20% of the world’s total rice production [27]. Nitrogen is one of the crucial and yield-limiting nutrients for rice and is closely related to the absorption of phosphorus and potassium [28]. Excess amounts of N cause a series of environmental problems such as soil quality degradation and excessive nitrate content in surface water and groundwater, which will seriously affect the sustainable use of farmland [29]. Both plant N uptake and N loss are affected by N concentration in the paddy floodwater and soil [30].

Overall, our study aims to address two important questions: (1) Can digestate addition increase rice yield? (2) What are the impacts of the combined application of digestate and straw on paddy soil and surface water nutrients? We explore the possibility for chemical fertilizer to be substituted by digestate partially or totally in rice production, providing suggestions for food security and sustainable agricultural development.

## 2. Materials and Methods

### 2.1. Experiment Treatments

Our field experiment was conducted for two years at the Suzhong Dadi Agricultural Technology Company in Gaoyou, Jiangsu Province, China. The experiment site is characterized by a subtropical warm monsoon climate, with an annual mean precipitation of 1000 mm and an annual mean temperature of 14.8 °C. The frost-free period lasts 217 days. The digestate was obtained from a local large-scale pig farm (Xingmu Pig Farm), with the following basic properties: pH was 7.76; total nitrogen (TN), available nitrogen (AN) and available phosphorus (AP) were 1012, 551 and 753 mg L^−1^, respectively. Six treatments (Table 1) were included in our study: without nutrients and straw (Control), digestate (D), digestate + fertilizer (DF), digestate + straw (DS), digestate + fertilizer + straw (DFS) and conventional fertilizer + straw (CS). For CS, the management was based on local farming habits. Each treatment had two plots (5 m × 4 m) as replicates. The irrigation and drainage time, frequency and quantity were identical for all treatments. Seedlings were raised by mechanical plastic plate and planted by artificial simulation machine at 18–20 days after seedling.

### 2.2. Sampling and Analytical Procedures

Plant and soil samples (0–20 cm) were collected from each plot at tillering, jointing, heading and mature stages. Soil samples were air-dried after plant residues and stones were removed. Plant samples were oven-dried at 105 °C for 30 min and then dried at 80 °C until a constant weight was reached. The N content in rice organs was determined by semi-micro-Kjeldahl method [31]. Soil catalase, alkaline phosphatase, urease and sucrase activities were determined using the permanganate titration method [32], disodium benzene phosphate colorimetry method, sodium phenoxide colorimetry and DNS colorimetry (3,5-initrosalicylic acid) [33], respectively. TN, AN, AP, AK and soil organic matter (SOM) contents in soil were tested by the semi-micro-Kjeldahl method, alkali disintegration spread–sodium bicarbonate method, sodium bicarbonate method [34], a flare photometer and potassium dichromate method [35], respectively. We randomly collected five water samples as replicates in each plot for detecting TN, NO_3_^−^-N and NH_4_^+^-N concentrations in paddy surface water at 1, 2, 3, 5 and 7 days after every application of digestate. TN content was measured with alkaline potassium persulfate [36], NO_3_^−^-N content was measured with UV spectrophotometry [37] and NH_4_^+^-N content was measured with Nessler′s reagent [38].

### 2.3. Statistical Analysis

Statistical analysis was performed using SPSS 16.0 Statistical Software (IBM, Chicago, IL, USA). One-way ANOVA was used to determine differences between treatments. Means were compared using least significant difference (LSD), and significance was determined at *p* < 0.05. Figures were drawn in OriginPro 8.5.1 (OriginLab, Northampton, MA, USA).

## 3. Results

### 3.1. Rice Yield and Plant N Distribution under Different Treatments

#### 3.1.1. Rice Yield and Yield Components

DFS had the highest ear number, which increased by 36.57% compared with Control and increased by 7.8% compared with DS. DF had the highest seed setting rate, with the value of 82.20%, increased by 15.47% compared with DS. DF had the highest yield, with the value of 9.86 t hm^−2^, while Control had the lowest yield, with the value of 7.06 t hm^−2^. The estimated yields decreased in the order of DF > DFS > CS > D > DS > Control. The rice yield under DF was increased by 39.66% compared to that under Control, and that under DFS was increased by 38.39%, while no significant differences were found for B and DS (Table 2).

#### 3.1.2. Plant N Distribution

**Figure 1:**
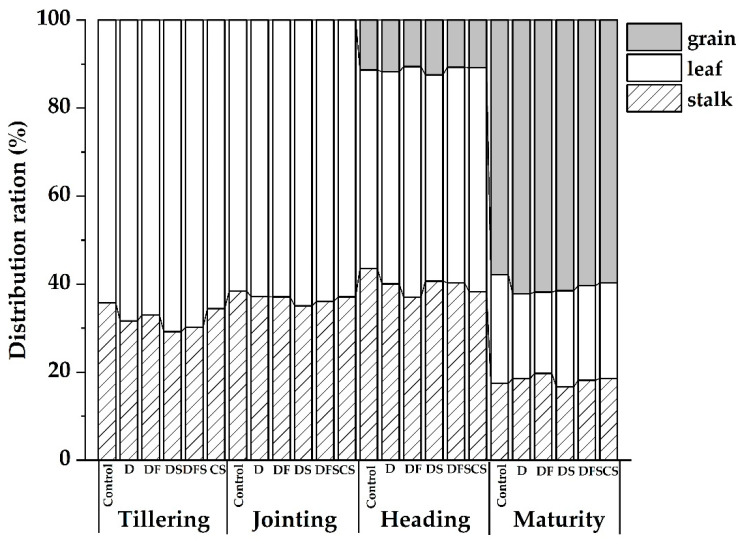
Plant N distribution under different treatments at the four growth stages.

In all treatments, N distribution in the stalk significantly increased from tillering to heading and decreased from heading to maturity, with translocation to grain occurring (Figure 1). At tillering stage, compared to the CS and Control, the digestate can promote the N distribution to leaf. At jointing stage, the trend of N distribution to leaf was the same as that at tillering stage, with DS being the highest and the control being the lowest, but the difference was not significant. The highest N distribution in leaf was found in DS both at tillering and jointing stages. At heading stage, N distribution to leaf was DF > CS > DFS > D > DS > Control; the highest value was 52.40% under DF, and the lowest was 45.08% under Control (Figure 1), with a significant difference, while the other four treatments had no significant difference. At maturity, N distribution to grain was in the decreasing order of D > DF > DS > DFS > CS > Control; the highest value was 62.18% under D, and the lowest was 57.87% under Control (Figure 1).

The results showed that when digestate was utilized as organic fertilizer, compared with conventional fertilizer, N distribution to leaf was higher at tillering stage, which laid a good foundation for growth at the early stage. More N was transferred to the grain at maturity, which had the advantage of N distribution to grain.

### 3.2. The Degree of Straw Decomposition

**Figure 2:**
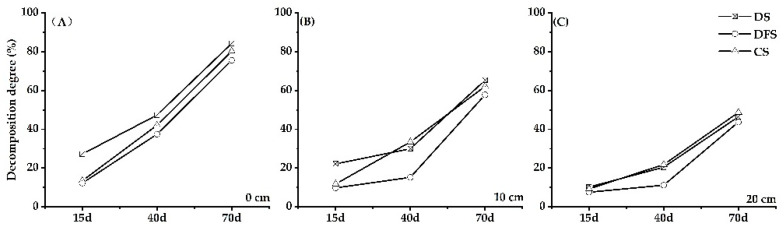
Dynamics of straw decomposition rate over time at 0 cm (**A**), 10 cm (**B**) and 20 cm (**C**) soil depths under DS, DFS and CS.

After the straw was buried, with the increase in soil depth, the decay degree of each treatment showed a downward trend. We found that on the 15th day after the straw was buried, the decomposition degrees at 0, 10 and 20 cm soil depth under DS were 2.02, 1.87 and 1.10 times higher than that under CS. The differences in decomposition rates between treatments decreased with the increase in soil depth (Figure 2). On the 40th day, the decomposition degree at 0 cm soil depth under DS was still higher than that under CS, while the difference was not significant at 10 and 20 cm soil depths. On the 70th day, the decomposition degrees at 0 and 10 cm soil depths under DS were just slightly higher than those under CS. The above showed that compared with chemical fertilizer, digestate had a better effect of promoting straw decomposition, especially at the early stage; when decomposing surface straw, this effect was more obvious.

### 3.3. Soil Enzymatic Activities

Soil catalase can catalyze the decomposition of hydrogen peroxide generated from the metabolism of the soil and living organisms into oxygen and water, so as to protect organisms from damages caused by hydrogen peroxide. Catalase activity was significantly higher at all periods under DS than under D. Catalase activity under DS increased by 8.46%, 8.72%, 7.69% and 8.29% at tillering, jointing, heading and mature stages, respectively, compared to that under B. Soil catalase activity under DS treatment increased by 11.57%, 27.17%, 21.76% and 14.28% at tillering, jointing, heading and mature stages, respectively, compared to that under CS (Table 3). Our results showed that the returning of digestate and straw could contribute to the improvement of soil catalase activity.

Soil alkaline phosphatase can catalyze the mineralization and hydrolysis of soil organic phosphorus and can help the plants to absorb phosphorous. During the tillering and jointing periods, alkaline phosphatase activity was significantly decreased in DS compared to D and in DFS compared to DF. At maturity, alkaline phosphatase activity in DS and DFS increased by 22.39% and 33.50%, respectively (Table 3). This indicates that the coupling of straw with digestate restrained the effect of alkaline phosphatase at the early growth stage but would contribute to the improvement of alkaline phosphatase activity at maturity.

Soil urease catalyzes the hydrolysis of amide N compounds into inorganic N compounds that can be directly absorbed by plants. There were no significant differences in urease activity at the early growth stage. At maturity, soil urease activity under DS was 25.65%, 32.03%, 34.13% and 48.90% higher than that under B, DF, DFS and CS treatments, respectively (Table 3). This indicates that the coupling of straw with digestate could improve the soil urease activity, especially at maturity.

**Figure 3:**
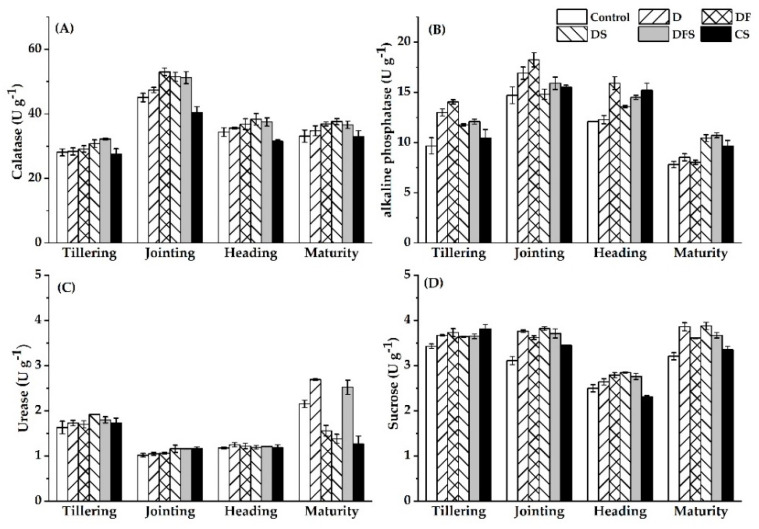
Soil catalase (**A**), alkaline phosphatase (**B**), urease (**C**) and sucrase (**D**) activities at the four growing stages. The error bars represent means ± 1 SE.

Soil sucrase plays an important role in the increase in soil soluble nutrients and is related to SOM, phosphorus, microbial number and soil respiration. Soil sucrase activity under B and DS was significantly higher than that under control at all periods; thus, either digestate alone or the combination of straw and digestate will contribute to the improvement of soil sucrase activity (Figure 3D).

### 3.4. Nutrients in Soil and Surface Water

#### 3.4.1. Soil Organic Matter and Nutrient Contents

**Figure 4:**
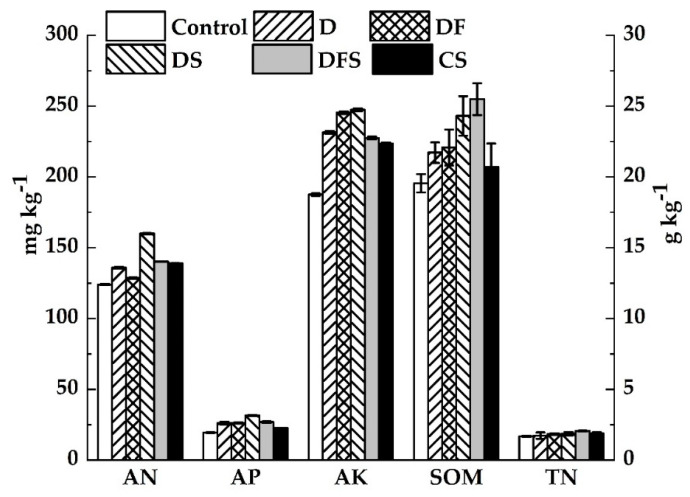
Soil organic matter (SOM), total nitrogen (TN), available nitrogen (AN), available phosphorus (AP) and available potassium (AK) contents at maturity under the different treatments. The error bars represent means ± 1 SE.

We found that the response of SOM and soil nutrients to treatments differed. The SOM decreased in the order of DFS > DS > DF > D > CS > Control at maturity (Figure 4). Concretely, the SOM under DS was 11.93% higher than that under B, and the SOM under DFS was 15.50% higher than that under DF (Table 4). The results showed that straw addition could benefit paddy soil quality after digestate application. The highest TN content was also found in DFS, increased by 12.02% compared with DF, and the TN content in DS was 8.14% higher than that in B.

The highest AN, AP and AK contents were all found in DS. The AN content in DS increased by 17.72% compared with D, and that in DFS was 9.04% higher than that in DF. The AP contents of D, DF, DS and DFS were 15.58%, 16.38%, 39.72% and 18.86% higher, respectively, than that under CS. Moreover, the AK content under DS was 10.71% and 31.90% higher than that under CS and Control, respectively (Table 4). Our results indicate that the digestate and straw combined application was beneficial to increase the nutrient content of paddy soil.

#### 3.4.2. TN, NO_3_^−^-N and NH_4_^+^-N Contents in Soil Surface Water

**Figure 5:**
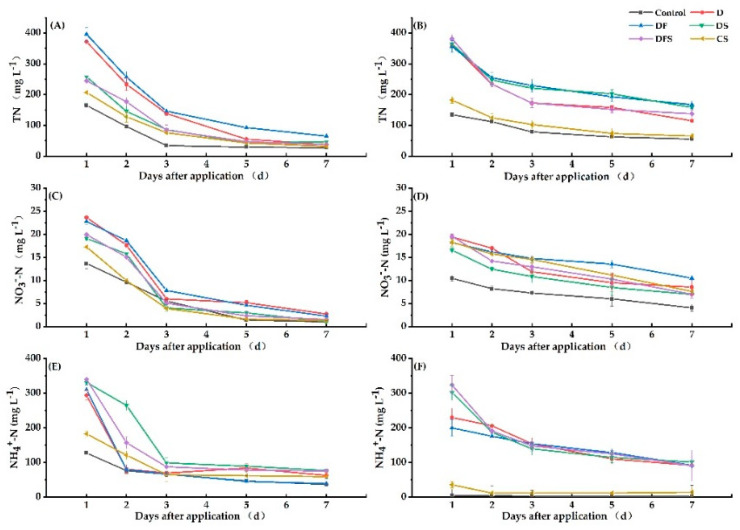
The TN (**A**,**B**), NO_3_^−^-N (**C**,**D**) and NH_4_^+^-N (**E**,**F**) concentrations in soil surface water during the 7 days after digestate was applied as base (**A**,**C**,**E**) and panicle (**B**,**D**,**F**) fertilizer. The error bars represent means ± 1 SE.

Our results showed that TN, NO_3_^−^-N and NH_4_^+^-N concentrations in soil surface water all decreased by over 50% in the first 3 days after digestate addition, regardless of treatments and applied periods (Figure 5).

When digestate was irrigated as base fertilizer, the TN concentration under D, DF, DS and DFS treatment decreased in the third day compared to that in the first day, and the decrease under DS and DFS tended to be faster than that under D and DF. Seven days later, the TN concentration under DS and DFS showed no significant difference with that under CS. This indicates that when digestate was irrigated as base fertilizer and coupled with straw, it would contribute to the absorption of N within 1–3 days. When digestate was irrigated as panicle fertilizer, the TN concentrations under D, DF, DS and DFS treatments within 1–3 days were significantly higher than those under Control and CS. On the 7th day, the TN concentrations of all treatments showed no significant differences, except DF was slightly higher (Figure 5A,B). Our results showed that after the digestate was irrigated as panicle fertilizer, despite being coupled with straw, the TN concentration was fairly high within 1–3 days; measures should be taken to avoid environmental water loss risk.

When digestate was irrigated as base fertilizer, the NO_3_^−^-N concentration under DF and DFS decreased by 65.44% and 74.65%, respectively, on the 3rd day compared to the 1st day. The NO_3_^−^-N concentration under DFS decreased faster than that under DF. When digestate was irrigated as panicle fertilizer, the NO_3_^−^-N concentration under DF and DFS decreased by 42.12% and 64.21%, respectively, on the 7th day compared to the 1st day. The NO_3_^−^-N concentration under DFS decreased faster than that under DF, similar to the situation where digestate was irrigated as base fertilizer (Figure 5C,D). This indicates that straw addition could definitely reduce the NO_3_^−^-N concentration of surface water.

When digestate was irrigated as base fertilizer, the NH_4_^+^-N concentrations of all treatments tended to become similar. Over time, they tended to decrease. On the 7th day, the NH_4_^+^-N concentration under DS and DFS was slightly higher than that under CS, but not significantly. This indicates that the NH_4_^+^-N concentration of surface water was high at the beginning but decreased to a relatively safe concentration after 7 days. When digestate was irrigated as panicle fertilizer, the NH_4_^+^-N concentrations under B, DF, DS and DFS on the 3rd day decreased by 32.99%, 22.92%, 53.77% and 54.27%, respectively, compared to those on the 1st day (Figure 5E,F). NH_4_^+^-N concentration under DS and DFS treatment decreased faster than that under B and DF. When digestate was irrigated as panicle fertilizer, coupling with straw could obviously reduce the NH_4_^+^-N concentration. Some NH_4_^+^ is oxidized to NO_3_^−^ in aerobic microsites in the soil surface water and is quickly lost by denitrification as it diffuses into anaerobic microsites [39].

## 4. Discussion

Our results show that higher N distribution in leaves at tillering stage under combined application of digestate and straw provided a good foundation for rice growth in the early stage. More N could be transferred to the grains at maturity, leading to better production [40], which was supported by the theoretical yields in our study. However, due to the very high input of chemical fertilizer, conventional management practice might still have higher rice yield than digestate input only, in agreement with previous studies [41]. In addition, digestate could promote straw decomposition based on our results, which supported that digestate addition in preprocess of straw decomposition might be the best promoter [42]. Our results show that the decomposition degree of DS was higher than that of DFS. This may be because higher rates of N fertilization inhibited soil enzyme activities and functional diversity of microbial communities [43]. Further efforts should be made to determine why negative effects appeared when chemical fertilizer was added. 

Our finding that digestate and straw combined application increased soil urease activity at maturity agreed with the results in rice–wheat and wheat–maize rotation systems in previous studies [44,45,46]. In addition, digestate application has been proved to increase the soil sucrase and phosphatase activities but significantly reduce the activity of soil catalase [47]. Our results suggest the same trends for sucrase and phosphatase activities during the key stages, though these trends were not significant; in contrast, digestate application likely increased catalase activity.

There are plenty of studies demonstrating that digestate application can increase not only SOM contents but also soil nutrients, such as TN, total phosphorus, AN and AP, with or without straw retention [48]. Concretely, we found that combined addition of digestate and straw caused a greater increase in SOM and nutrient (i.e., TN, AN, AP and AK) contents than digestate applied alone. This could possibly be explained by digestate being characterized as rich in nutrients and thus being able to supply the nutrients directly; it could also increase soil nutrients indirectly through promoting enzymatic activities [49]. In agreement with previous studies, N concentration in soil surface water increased after using digestate as base fertilizer or panicle fertilizer in this study [50,51]; however, the greater differences in TN, NO_3_^−^-N and NH_4_^+^-N concentrations between treatments after digestate addition as base fertilizer compared to digestate addition as panicle fertilizer were likely the result of N saturation in soil surface water. Furthermore, after using digestate as base fertilizer, NO_3_^−^-N contents under D and DF were higher than those under DS and DFS, but the NH_4_^+^-N contents under D and DF were lower than those under DS and DFS. This might because that NO_3_^-^-N mainly came from the nitrification of NH_4_^+^-N [52]. When straw decomposed, oxygen was consumed, thereby inhibiting nitrification and promoting denitrification. 

## 5. Conclusions

Digestate addition maintained the rice production and had some positive effects on paddy soil and water properties in our study. Digestate application significantly increased rice yield compared with Control; combined with straw addition, it could distribute more N to rice grains, which benefited rice production. Moreover, digestate promoted straw decomposition compared with CS. Activities of catalase, urease, sucrase and phosphatase at maturity under DS were all higher than those under Control and CS. Thus, among the six treatments, DS is the best application. Above all, our findings could provide some evidence for the possibility of using digestate to replace chemical fertilizer in rice production. We also suggest that further efforts should be made to explore the mechanisms of the combined application of digestate and straw so that suggestions can be provided for the better management of digestate applied in the future. 

## Figures and Tables

**Table 1:** Specific instructions of six experimental treatments.

Treatments	Basal Fertilizer		Tillering Fertilizer	Jointing Fertilizer	Panicle Fertilizer		Straw Incorporation Rate (t hm^−2^)
	Digestate (t hm^−2^)	Compound Fertilizer (kg hm^−2^)	Urea (kg hm^−2^)	Urea (kg hm^−2^)	Digestate (t hm^−2^)	Urea (kg hm^−2^)	
Control	\	\	\	\	\	\	\
D	120	\	\	\	90	\	\
DF	120	\	211.5	\	90	\	\
DS	120	\	\	\	90	\	7.5
DFS	120	\	211.5	\	90	\	7.5
CS	\	525	211.5	130.5	\	64.5	7.5

**Table 2:** Rice yield and yield components under different treatments.

Treatments	Ear Number (10^4^ hm^−2^)	Grains per Spike	Seed Setting Rate (%)	1000-Grain Weight (g)	Theoretical Yield (t hm^−2^)
Control	273.38 ± 13.32 c	115.49 ± 12.12 b	79.64 ± 0.10 a	28.06 ± 1.08 a	7.06 ± 0.06 d
D	293.06 ± 19.98 c	147.82 ± 12.37 a	75.48 ± 0.05 b	26.86 ± 0.34 c	8.78 ± 0.67 c
DF	346.32 ± 23.64 b	126.79 ± 3.65 b	82.20 ± 0.07 a	27.30 ± 0.55 b	9.86 ± 0.60 a
DS	339.66 ± 19.98 b	131.71 ± 1.24 ab	71.19 ± 0.02 c	27.39 ± 0.28 b	8.72 ± 0.97 c
DFS	373.35 ± 6.66 a	122.62 ± 7.95 b	76.68 ± 0.02 b	27.83 ± 0.03 a	9.77 ± 1.18 a
CS	368.32 ± 26.63 a	120.72 ± 17.14 c	77.17 ± 0.07 b	27.79 ± 0.01 b	9.53 ± 1.60 ab

Different letters (a, b, c, d) indicate statistically significant differences between treatments at *p* < 0.05 (honest significant difference (HSD) test).

**Table 3:** Soil catalase, alkaline phosphatase, urease and sucrase activities at the four growing stages under different treatments.

Enzymes	Treatments	Tillering	Jointing	Heading	Maturity
Catalase (Ug^−1^)	Control	28.08 ± 1.06 b	45.06 ± 1.33 c	34.34 ±1.35 c	33.09 ± 1.88 c
	D	28.37 ± 1.12 b	47.36 ± 0.84 b	35.59 ± 0.35 b	34.74 ± 1.55 b
	DF	29.12 ± 2.01 b	52.99 ± 2.19 a	36.80 ± 1.65 ab	36.87 ± 0.60 a
	DS	30.77 ± 1.16 a	51.49 ± 1.40 a	38.33 ± 1.80 a	37.62 ± 0.93 a
	DFS	32.19 ± 0.38 a	51.21 ± 1.85 a	37.49 ± 1.29 a	36.60 ± 1.14 a
	CS	27.58 ± 1.64 c	40.49 ± 1.73 d	31.48 ± 0.52 c	32.92 ± 0.90 c
Alkaline phosphatase (Ug^−1^)	Control	9.67 ± 0.81 c	14.70 ± 0.84 b	12.10 ± 0.03 bc	7.81 ± 0.31 b
	D	12.99 ± 0.36 a	16.92 ± 0.61 a	12.28 ± 0.41 b	8.53 ± 0.36 b
	DF	14.05 ± 0.21 a	18.24 ± 0.71 a	15.91 ± 0.63 a	8.03 ± 0.21 b
	DS	11.77 ± 0.11 ab	14.80 ± 0.51 ab	13.59 ± 0.11 ab	10.44 ± 0.35 a
	DFS	12.08 ± 0.24 ab	15.91 ± 0.61 a	14.50 ± 0.21 a	10.72 ± 0.23 a
	CS	10.47 ± 0.83 b	15.51 ± 0.20 ab	15.20 ± 0.71 a	9.66 ± 0.56 a
Urease (Ug^−1^)	Control	1.63 ± 0.14 b	1.02 ± 0.04 c	1.18 ± 0.02 ab	2.15 ± 0.38 cd
	D	1.73 ± 0.06 b	1.05 ± 0.03 b	1.25 ± 0.05 a	2.69 ± 0.22 b
	DF	1.70 ± 0.08 b	1.06 ± 0.02 b	1.22 ± 0.06 a	2.56 ± 0.12 b
	DS	1.92 ± 0.01 a	1.16 ± 0.08 a	1.19 ± 0.04 ab	3.38 ± 0.10 a
	DFS	1.80 ± 0.07 a	1.16 ± 0.01 a	1.21 ± 0.01 a	2.52 ± 0.16 b
	CS	1.73 ± 0.11 ab	1.17 ± 0.03 a	1.19 ± 0.06 ab	2.27 ± 0.17 c
Sucrase (Ug^−1^)	Control	3.43 ± 0.05 b	3.11 ± 0.39 c	2.50 ± 0.08 b	3.21 ± 0.28 bc
	D	3.67 ± 0.20 a	3.76 ± 0.33 a	2.64 ± 0.07 a	3.86 ± 0.29 a
	DF	3.73 ± 0.29 a	3.62 ± 0.14 ab	2.79 ± 0.06 a	3.61 ± 0.01 ab
	DS	3.64 ± 0.01 a	3.82 ± 0.04 a	2.85 ± 0.01 a	3.88 ± 0.18 a
	DFS	3.65 ± 0.05 a	3.71 ± 0.10 a	2.76 ± 0.07 a	3.67 ± 0.26 ab
	CS	3.81 ± 0.10 a	3.45 ± 0.01 b	2.31 ± 0.13 b	3.35 ± 0.18 b

Different letters (a, b, c, d) indicate statistically significant differences between treatments at *p* < 0.05 (honest significant difference (HSD) test).

**Table 4:** Soil organic matter (SOM), total nitrogen (TN), available nitrogen (AN), available phosphorus (AP) and available potassium (AK) contents at maturity under the different treatments.

Treatments	SOM (g mg^−1^)	TN (g mg^−1^)	AN (mg kg^−1^)	AP (mg kg^−1^)	AK (mg kg^−1^)
CK	19.54 ± 0.65 c	1.68 ± 0.03 c	123.90 ± 5.40 c	19.38 ± 0.22 d	187.50 ± 9.85 c
D	21.71 ± 0.73 b	1.72 ± 0.23 c	135.82 ± 5.61 b	26.04 ± 1.02 b	231.37 ± 5.98 b
DF	22.07 ± 1.26 b	1.83 ± 0.06 b	128.48 ± 2.42 c	26.22 ± 0.51 b	245.33 ± 7.88 a
DS	24.30 ± 1.39 a	1.86 ± 0.11 b	159.89 ± 2.39 a	31.48 ± 0.32 a	247.32 ± 9.97 a
DFS	25.49 ± 1.13 a	2.05 ± 0.03 a	140.09 ± 6.20 b	26.78 ± 0.55 b	227.38 ± 5.98 b
CS	20.71 ± 1.65 b	1.88 ± 0.08 b	138.94 ± 5.24 b	22.53 ± 0.25 c	223.39 ± 8.96 b

Different letters (a, b, c, d) indicate statistically significant differences between treatments at *p* < 0.05 (honest significant difference (HSD) test).

## Data Availability

No new data were created or analyzed in this study. Data sharing is not applicable to this article.
